# Modern Metaphysics
*Philosophy of Spirits in relation to Matter. By C. M. Burnett, M.D. 1850. (Second Notice.)


**Published:** 1851-07-01

**Authors:** 


### Art. II.—MODERN METAPHYSICS.*

In continuing our analysis of Dr. Burnett's work, we would observe that he does not limit the operations of the "spirit of life" exclusively to vitality, using this term in its ordinary acceptation; but, by a fallacious method of induction, endeavours to resolve those attributes and phenomena, hitherto regarded as instinctive and mental, into a *positive* dependence on ONE common principle. By this philosophy the

* Philosophy of Spirits in relation to Matter. By C. M. Burnett, M.D. 1850. (Second Notice.)

psychical superiority of man, compared with the brute creation, is to be referred solely to organic structure co-operating with the "spirit of life." Pursuant to this notion, the author remarks:—

"The organization of the brain being fixed in the number and size of its various parts, transmits the mental phenomena accordingly in a more or less elaborate manner. This certainly favours the idea, that if there be any difference in the immaterial cause of the vital and mental phenomena of animals, it is only a difference of degree in the organic parts; for the one is so intimately mixed up with, and dependent upon the other,—they are so essentially the same in some of their effects, while they are in operation so inseparable, that they cannot be said to have two distinct spirits for their origin. Indeed, if we separate mind from life in its true efficient cause, we may with as much reason separate the efficient cause of motion and sensation."—p. 154.

The analogy here drawn between the cerebral apparatus of man and animals with the view of supporting the hypothesis that instinctive manifestations are dependent on the same principle, is erroneous and absurd; at the same time we admit, that we owe to physiology the discovery that a refined organization is the instrument which the mind employs in holding communication with external objects; and also the fact that the psychical phenomena of animals increases in a ratio with the development of a complex brain and nervous system. We know that in the butterfly the brain is much increased in size as compared with that of the caterpillar, the visceral nerves dwindling in proportion to the development of those of active life and volition; that the same fact is largely exemplified also by a comparison of the nervous system in every class of animals, from molluscs up to man; and yet we must use comparative physiology with the greatest circumspection, because the psychology of animals recognises a principle of intelligence radically different from that of man.

In man the hemispherical ganglia of the brain are positively larger in proportion to the rest of the brain than in any other animal, not excepting the elephant. This superiority is chiefly manifested in the size, number, and direction of their convolutions; thus, Cuvier has very properly removed man from the rank in which Linnæus placed him, as the associate of apes and monkeys, and left him alone at the head of the creation. For characteristic differences are found to exist between the brain and nervous system of man, and those of the higher Simiæ. Mr. Owen informs us, that though the brain of the chimpanzee approaches nearest to the human brain, yet it differs "in *the flatness of the hemispheres*, in *the comparative shortness of the posterior, and in the narrowness of the anterior lobes*."

The conformation of their respective skulls attests the same fact. When we leave the vertebrated animals, the cerebral system is less and less developed; and in correspondence with this change, to us a blind instinct more or less supplants that pseudo-intelligence which becomes manifest under the conditions supplied by a higher development of the nervous system.

Those creatures whose nervous system is purely ganglionic, and presides more especially over the instinctive organs and the involuntary motions, though not perhaps wholly devoid of this pseudo-intelligence, are endued with much less of it, while their instinctive operations are all but miraculous.

It is evident, therefore, from these and other facts which may be adduced, that physiological inquiries do not enable us to identify the pseudo-intelligence that accompanies the instinct of animals, with that which in man springs from the existence of an incorporated spirit.

The nature and habits of animals are found greatly to vary, even when their cerebral structure very closely resembles each other; as in the case of the wolf and the dog, the hare and the rabbit, and in other instances. Nor are the variations that exist within the limits of an uniform type of nervous structure, as in birds, sufficient, without the aid of discriminative reasoning, to explain the amazing diversity of habits and instincts of the different species. "The organized world," observes Dr. Prichard, "possesses no greater contrasts and resemblances than those which we discover in comparing mankind with the inferior tribes. That creatures should exist so nearly approaching to each other in all the particulars of their physical structure, and yet differing so immeasurably in their endowments and capabilities, would be a fact hard to believe, if it were not manifest to our observation. The *differences* are everywhere striking; the *resemblances* are less obvious in the fulness of their extent."

There are, however, instances in the case of animals, in which similarity of structure has its influence; thus, the ostrich family bears some resemblance in structure to the mammalia, and their instincts so agree, that the former actually associate with some of the latter, particularly the quagga and zebra. Nevertheless, instinct is the chief instrument by which Providence preserves the different races distinct, their habits being commonly so dissimilar as to be a hindrance to their association.

But the error committed by Dr. Burnett in confounding instinct with an intelligent entity, is comparatively unimportant, compared with that of resolving the phenomena of each into absolute dependence on cerebral structure. The one exists under the supreme domination of a law that will expire when the cycle of existence has terminated; while the other possesses a positive being "that once was not, but shall never cease to be."

That we do not misapprehend Dr. Burnett's views on this subject, will be obvious from the following passage:—

Dr. Burnett observes, "In animals we notice mental phenomena which decide without a doubt that they possess a power of perception, of memory, of judgment, of will, of attention; that they have thoughts and desires and mental operations, allied in character, so far as they go, to those in man. Moreover, the chemical analysis of the cerebral matter in animals shows that the phenomena of mind in all must proceed from the operation of a *similar spirit;* for there is no material difference in the component material elements."—p. 156.

We cannot concur with views so utterly at variance with the psychology and physiology of the subject; at the same time we feel it right to observe that Dr. Burnett is not the only writer who has taken this view of the subject. Locke considered instinct to be an attribute of the soul of brutes. He says, "that animals do, in certain instances, reason; as they have sense." Dr. Hancock takes the same view, and adopts the same language. Dr. Good admits that reason is united in brutes with sensation and instinct. Others entertain the opinion that instinct is the result of the direct action of the Deity on the sentient nature of brutes. Addison held this view of the subject, and asserted that "God is the soul of brutes." We need scarcely say that such an opinion is disproved by the operation of instinct itself. For example, would it be possible for the flesh-fly to mistake the blossom of the carrion plant, as often happens, for a piece of flesh, and lay her eggs in it, if God were its immediate instructor? Or would the hampster break the wings of *dead* birds, as well as *live* ones, to prevent their escape? Or should we see, as we sometimes do, one instinct clashing with another in the same individual? Thus, the migratory instinct of birds is sufficiently strong to overcome the force of parental affection, when an unfavourable season and other causes have occurred to retard the maturity of the brood; and desertion is the consequence. Hence, the swallow often leaves its later brood to perish in the nest. The sea-fowl of Flamborough Head presents us with another example.

The determinateness and perfection of the movements of instinct in general, independently of instruction or experience, strikingly contrast with the operations of reason.

"Reason PROGRESSIVE, instinct is COMPLETE."—YOUNG.

We therefore conceive that it will not be difficult to ascertain, that *reason* is different from *instinct* in its SOURCE, in its NATURE, in its OPERATIONS, and in its END.

Dr. Burnett, in conformity with a previously established theory resolves instinctive and mental phenomena into absolute dependence on organization, and what he pleases to denominate a "spirit of life." Thus he observes:—

"In animal life, ideas, and thoughts, the result of a mixed sensation, or of a more varied display of the abstract power of sensation, are superadded by means of a cerebral apparatus to simple sensation, which alone takes place in vegetable life. So that the distinction between the sensation of vegetable life, and that which forms the basis of the mental operations of animal, is apparently one of degree."—p. 138.

Here, the "spirit of life," like a chain, is supposed to run through nature; connected by links, which lay hold of each other, are the intermediate steps between substances which differ in their qualities. Instinct at one point embraces and becomes a low degree of reason; in the other it sinks and is lost in the appetites and passions. But dissimilar principles cannot correspond; mind cannot partake of the properties of matter; no genus, or even species, glides imperceptibly into one another.

If the "spirit of life," co-operating with the spirits of heat and electricity, form organized beings having instinct and reason, then mind must partake of the properties of those elements on which its existence is said to depend; must be, like them, material and obvious to the senses.

The spirit which animates, and the inorganic elements which constitute an oak or a cedar, the highest order of vegetables, alike with that which animates and forms the substance of a worm or a polypus, the lowest in the scale of animated beings, must be the same as that which puts together the fabric of man, and on which the existence of mind depends.

By this the apostrophe of Shakspeare is mere rhapsody, when he says—"What a piece of work is man! How noble in reason! How infinite in faculties! In form and moving how express and admirable! In action how like an angel! In apprehension how like a god! The beauty of the world! The paragon of animals!"

Or that of Young:—

"How poor, how rich, how abject, how august,
How complicate, how wonderful is man."

Thus, the hypotheses of Dr. Burnett destroys, with sweeping effect, the great features by which man is contradistinguished from other beings of the creation, allies him in inseparable connexion to the worm that crawls beneath his feet, to the animals that roam, and to the cedar that inhabits the forest.

But nature has no points of an equivocal character. No specimen is found in any cabinet of a mineral in a state of transmutation, passing from iron to gold, or rising into vegetable organization; a distinction in principle is maintained by a distinction in operation. By the supposition that the spirits of heat, electricity, and life, run through created nature, the clod of the valley, and the plant it nourishes, the movements of a worm, and the mind of man, are identified in the essential nature of their existence; thus life is at some point obscured and lost in inert matter; a supposition contrary to the whole analogy of nature. But Dr. Burnett observes, that—

"The difference between what is called instinct in animals and reasoning in man, depends entirely upon the fact of the number of these attributes of the mind in the latter being added to by these attributes of prescience and conscience, and so being numerically greater than they are in the former."—p. 178.

An hypothesis at once contravened by the evidence of instinct, as seen in operation. An attribute or faculty pre-supposes the existence of something on which it depends; it can, therefore, have but a relative existence. A faculty or an attribute is subject to declension and change, being derived from a cause foreign to itself, and, therefore, cannot constitute instinct, cannot be like it—a power ever perfect and complete.

Instinct cannot be cultivated; reason may be. Instinct is perfect and irresistible at its commencement, as has been observed; thus, a young bee betakes itself to the complex operation of building cells with as much skill as the oldest of its compatriots. The brood of young ducks brought up under a hen, the moment they see the water plunge at once into their native element, in contempt of all previous training and example. Have we occasion to ask whether they reason on their possession of a web foot, or whether they rush into the water in obedience to an impulse which guides their movements? Mark the unerring, yet compulsory, choice with which the moth places her eggs upon food which she herself can never use. The conduct of the nut-weevil again illustrates the same wonderful instinct, which provides the fitting nutriment for its offspring. We cannot, therefore, infer, from these examples, that an intelligent principle, corresponding in its nature to that possessed by man, dictated them.

If Dr. Burnett's views be correct, this must be so. Thus, at every step, a survey of the operations of instinct must demonstrably show that an obvious distinction must exist between it and the principle on which human intelligence depends.

If they be identical in their nature, how is it that the young of every kind possess a knowledge of the peculiar powers that are to appertain to them hereafter, even before the full formation of the organs in which these powers are to reside, while example and instruction are so lavishingly required with the young of our species?

This truth of natural history is well expressed by Lucretius:—

> "The young calf, whose horns
> Ne'er yet have sprouted, with his naked front
> Butts when enraged; the lion whelp or pard
> With claws and teeth contends, ere teeth or claws
> Scarce spring conspicuous; while the pinioned tribes
> Trust to their wings, and from the expanded down
> Draw, when first fledged, a tremulous defence."

Instead, therefore, of attempting to explain the nature of instinct by a physiological or psychological comparison, which cannot for a moment be entertained, we think that it may rightly be considered a law; a law of impulse, adapted, for the most part, to the well-being and propagation of the animal creation.

The supposition that instinct consists in the exercise of certain attributes or faculties destroys the idea of a law; for a faculty has no direct and immediate governing power: it is itself dependent, and has but an imperfect influence. There can be no principle in nature independent of its laws, from which instinct issues, as reason does, from the mind.

Instinct is never weary; and can it, therefore, be imagined that any dependent or secondary power can be competent to its office? It is a power emanating from the Creator, and not a secondary influence, which instructs the swallow and other birds when to migrate. If it be instinct, can it be less than a law? "The stork in the heaven knoweth her appointed times; and the crane and the swallow observe the time of their coming."

The impulse by which the vernal immigrants are impelled to undertake an autumnal journey is observed in birds long subject to the unnatural restraints of the cage; exciting in them urgent signs of restlessness at the accustomed time of departure. The migration of the cuckoo is wholly independent of example or instruction, for it has never known a cuckoo's parental care.

But Dr. Burnett attributes this extraordinary exponent of a law to the mental endowments which those birds possess. Thus, he observes:

"The power to find the longitude, which many sea-birds possess, if it were moved by the high attributes of man, would be a more wonderful power than any he possessed. But it acts irrespective of prescience, and is effected through the agency of those attributes I have named."—p. 179.

If we admit animals to be endowed with the attributes which Dr. Burnett supposes, we make them responsible for their actions, not only to man, but to their Creator: a supposition which has not the slightest foundation.

The swallow is as irresponsible as a block of marble; for to whom does it give an account of what it does? Warned by an irresistible impulse it departs, and by the same impulse returns, with a regularity unknown to the seasons : they vary, but the swallow returns on the same day in every succeeding spring. The power, be it a law, or be it not, is uniform, like that which rolls the earth along its path. And wherever uniformity can be predicated of any fact, that fact assumes the character of a law. But Dr. Burnett asserted that animals possess, and that their actions are influenced by, the attributes which can alone apply to an immaterial entity.

"Believing," he observes, "that there are nevertheless certain primary and independent attributes in the mind of animals as of man, I think one of those is memory, another attention, a third comparison, and a fourth will."

It certainly appears difficult to draw the line of demarkation between that kind of intelligence which accompanies instinct, and that intelligence which constitutes the proof of the existence of an immaterial entity, which is denominated *mind:* but we think that we shall be able to show that instinctive action is founded upon impulse, and that which concerns the operations of reason upon reflection.

Those singular facts of natural history to which we have adverted, prove that instinct acts under the dominion of impulse. If we regard instinct as an active principle (and without activity it sinks into a mere aptitude or capacity), it is impossible that we can detach it from its dependence upon those impulses by which it is rendered visible. That instinctive activity is not invariable in any of its modes, is demonstrated by fact; and hence the conclusion is evident, that activity is not essential to the aptitude or capacity of such creatures as act solely under the dominion of impulse. It therefore follows, that the activity of instinct must be derived, and derived from those impulses which the creature invariably obeys when they operate upon its natural capacity, either through the medium of the senses, or without their instrumentality.

By the employment of the phraseology "primary and independent attributes in the mind of animals," Dr. Burnett lapses into the error of confounding ideas which the form of expression makes distinct. No attribute can be primary and independent;—an attribute implies the existence of something of which it is the attribute or quality, while the terms, "primary and independent," pre-suppose an abstract positive existence. An independent attribute is, therefore, a contradiction. It is evident, too, that Dr. Burnett has, by the employment of those terms which characterize the operations of mind, regarded instinct, for the most part, as acting under the influence of intelligence, and not that which acts under the guidance of a blind impulse.

We deny, however, that instinctive actions take place under the guidance of the attributes—memory, judgment, and will. An instinctive action can have no existence unless foreign causes impel; it is evident, therefore, that though the aptitude or capacity may be essential to the creature, the activity is not; and it is in relation to the propensity in action, and not to the mere capacity, that instinct is here considered.

Now, as instinct acts under the dominion of impulse, it is absurd to imagine that it has any independent existence; for, if this had been the case, it would as fully exist when these causes ceased to impel, as when they operated in all their vigour. But this we know is contradicted, both by experience and observation. Of the fact, that instincts may slumber for generations, we mention that which leads hive-bees to set about rearing a new queen, when their former sovereign is by accident destroyed. It may not have occasion to be called into action during a long series of generations in a hive; and yet, the moment the extraordinary occasion demands it, it is ready to be developed.

Dr. Burnett states that "animals possess judgment" (a primary and independent attribute, too, according to his view of it), "and that their actions are influenced by it." That instinct, in what light soever it may be viewed, can have no elective or counteracting power, is evident from its dependence upon impulse for all its energies.

The exercise of an elective power can never be reconciled with the influence of impulsions which cannot be resisted; because, both being independent, nothing can command their mutual co-operation. If, therefore, we attribute to instinct an elective power, and yet retain in our minds that idea of its dependence which we have been endeavouring to establish, we shall be compelled to allow that it is dependent upon impulse, and yet independent of it at the same time.

By this reasoning, the province of instinct, as well as its nature, is marked out. It presents itself to our view by certain characteristics with which it cannot dispense, and appears within specific boundaries, beyond which it cannot pass. We behold it acting under the dominion of impulsive causes, and perceive its dependence on them so clearly, as to exclude from its nature every property of a constant and independent existence. The "primary and independent attributes," therefore, which Dr. Burnett believes to exist in animals, apart from the contradiction which that supposition involves, can have no relation to the nature of instinct.

But reason is clearly distinguished from instinct, not merely as one faculty differs from another, but as containing a power by which it can withstand and counteract instinctive impulse. This is a power to which mere instinct is incompetent.

Hence philosophy and common sense show, that as brutes are unable to withstand the influence of those causes from which they act, they must be destitute of that principle to which the name of reason has been given; and, consequently, by being destitute of its power, they must submit to impulsive force.

Indeed, if reason were not radically different from instinct, man could not have possessed a moral nature. Thus reason differs from instinct in its source and nature, as well as in its operations and end. If then, "reason stands for a faculty in man, that faculty whereby he is supposed to be, and actually is, distinguished from beasts, and wherein it is evident he far surpasses them," as Locke asserts, are not our views carried to an immaterial substance as that which alone can be its primary source?

In the general operations of reason (for we purposely adhere to a term having a specific meaning with respect to man,) are included the attributes which Dr. Burnett states are individually connected with the acts performed by animals. Hear his words:—

"The mental operation that takes place so that they may act according to circumstances which lead to their benefit or their injury, is an operation effected simply by the aid of the attributes of attention, memory, and comparison, acting upon stronger feelings. Thus, an animal will avoid that food that is noxious, not because it knows the future consequences of again partaking of it, but simply because its attention, memory, and judgment, apprize it of the former effects the noxious food had upon it."—p. 179.

Instinct being *special* in its object, while reason is *general*, it is evident that the illustration, given by the author, disproves the assumption that animals are endowed, or that any of their actions are directly influenced by those attributes which apply only to the operations of reason.

The attribute of judgment can have no place without the existence of reason. It is on the comparisons which reason deliberately and freely makes that the decisions of judgment are founded.

It is absurd to suppose that an "animal refuses noxious food, because its judgment apprizes it of the former effects the noxious food had upon it;" for *no* animal will, voluntarily, take food that is noxious; the impulse under which it acts, being always for its own preservation.

*Judgment* implies objects of comparison; a perception of the agreement or disagreement of ideas; an attribute which characterises the operations of an intelligent entity, and not one which brutes possess.

Further, reason is distinguished in its nature from instinct by the power which it exercises in tracing the ideas presented to it, through all their labyrinths, previously to the adoption of any of them. The past, the present, and the future enters into the active operations of reason.

But, as neither of them can possibly be included in our idea of instinct, we are here presented with a specific difference in the *nature* of these intellectual and animal powers.

We know also, that as reason, in its operations, calculates upon consequences remote from that inducement which first excited its attention, and with which that inducement has no apparent connexion, reason must in this case operate in a region, within the confines of which instinct can never enter; and this leaves us in the possession of a decisive difference between their respective OPERATIONS and POWERS.

Man differs from the brute creation in his possession of a moral nature, moral powers, and a moral sense.

In some of them, indeed, we perceive a degree of sagacity which, at times, half induces us to associate the instinctive with the rational faculties; but in none, not even the most intelligent, have any vestiges or symptoms of a moral nature, of moral powers, or of a moral sense, ever yet been discovered.

The rational and intellectual faculties must submit to the dominion of a *moral instinct;* and in all cases where moral good and evil are concerned, they dare not encroach upon her province. It is on this principle that one great end of reason is, to regulate the solicitations of desire, and to render appetite subservient to propriety, and the principles of immutable justice, while the great end of instinct is to obtain gratification, independently of moral right and wrong.

The intelligence of man exceeds that of animals in a *sciential,* as well as in a moral direction. Man is distinguished from the brute creation by the faculty of language, and by *his yearning after immortality.*

From the exposition we have given of Dr. Burnett's views of *instinct,* it might be anticipated that his opinions on *mind* would be equally inconclusive and absurd, and they are so.

He denies to mind a positive existence, affirming that it "*cannot be an entity,*" and endeavours to show that this principle of our constitution is simply a "mode of action;" an aggregation of certain "*qualities and powers;*" that a distinction exists between "the attributes of the mind and the intellectual faculties and social feelings;" that man is contradistinguished from animals so far as the existence of mind is concerned, solely by the attributes of "*prescience* and *conscience;*" that the mind is worked by the agency of powers and attributes that are independent of the faculties and feelings;" that the faculties and feelings have their root in the sentient power of the nervous system, while the attributes of the mind have their root in the motive power of that system;" that we err in making consciousness a mark of distinction by which to recognise spirit, &c.

But contrary to these undefinable opinions, and, we think, somewhat incomprehensible too, except to the author, we maintain that mind is an entity whose operations, as manifested in thought and consciousness, enable us to submit it to an analysis equally rigid to that of any branch of physical science.

Metaphysicians have not, we think, generally treated their subject sufficiently as analysts; they have not, it appears, established a science of mind impregnable to the assaults of mere abstract views, or we should surely never have to subject such doctrines as those propounded in the work now under our consideration to a metaphysical analysis.

We are acquainted with but two primary substances that have in themselves a positive existence, and these are contradistinguished from one another by the terms *matter* and *spirit.* The human mind is that spirit alone of which we can be said to have any immediate knowledge.

Whatever has any positive existence must be material or it must not; and that which has any existence, and is not material, must be immaterial; there can be no medium between these two extremes. Not only so, but whatsoever has a positive existence, must have existed antecedently to our apprehension of it; because it, in its very nature, supposes the pre-existence of that which is apprehended; for, to suppose it possible for us to have an apprehension of what had not a previous existence, is to suppose we can apprehend what has no existence; which includes this contradiction, that we can apprehend that of which it is impossible for us to have the most distant apprehension.

The dignity and importance of this subject rests altogether on the truth of the *positive existence of mind.* Of this, Dr. Burnett seems to be aware.

"It is a point of great importance," he remarks, "to determine whether the mind, which includes the whole aggregate of the intellectual faculties, sentiments, feelings, and propensities, *has any real existence* as an *entity or not.*"—p. 157.

It is, however, illogical, on the part of the author, to raise the question, and decide it in the same words. The collocation of words, "to determine whether the mind" "has any real existence as an entity or not," is periphrastical and misleading. So, also, that of "real existence," joined to the word "entity:" both forms of expression implying the idea of positive existence. It is the same with the phraseology, "mind and intellectual faculties." If the mind includes an aggregate of the intellectual faculties, it must necessarily include an aggregate of its own faculties; for who ever conceived the idea that the intellectual faculties were other than the faculties of mind.

The following references from the work before us will define more clearly the author's views on this fundamental doctrine of metaphysics.

"If we analyze the different parts or properties of what is called mind, whether in man or in animals, we shall find it to be composed of certain powers and qualities, which, in the aggregate, are comprehended in the term mind."

"In contemplating what we call the mind, in animals and in man, we discover it to be composed, first, of a series or collection of faculties intended to give certain and distinct information as to the nature and the uses of objects around us."—p. 162.

"It has been shown that mind, whether in man or in animals, cannot be an entity."—p. 166.

"The individual intellectual faculties may be engaged separately from each other, and from the social faculties."—p. 183.

"One reason for supposing these attributes to be separate is, that it is nowhere proved, though they have power to act in unison, that they possess any real power over each other."—p. 185.

We gather from these observations, that this "point of great importance," viz., "whether the mind has any real existence as an entity or not," is decided in the negative, without a single argument to justify an opinion so dangerous and absurd.

An "aggregate," or "collection of faculties," sentiments, feelings, and propensities, pre-supposes the abstract existence of each faculty, sentiment, &c., a supposition which, by destroying any idea of the *positive existence of mind*, involves us in a chaos of contradictions, and a labyrinth of absurdities.

How can the *mind* be made up of the feelings and propensities? qualities purely animal, and which, therefore, cannot enter into an abstract consideration of a mental principle.

The "attributes," which Dr. Burnett informs us have a separate or distinct existence, are "attention, perception, memory, comparison, will, prescience, and conscience."—p. 176.

This absurd opinion takes away the very qualities by which alone an intelligent entity can be known to exist.

It is absolutely impossible to form any idea of *mind* abstracted from perception, judgment, will, consciousness, &c.; for, when the only properties are destroyed which contra-distinguish this entity from a material one, there is an end to all evidence in support of the mind's existence. It is only from the manifestation of intelligence that we can have any evidence of its existence. That of which we are in ignorance we cannot pronounce to have a being.

By the supposition that consciousness, will, perception, &c., exist apart from a conscious and perceptive substance, the author has necessarily involved himself in the admission of absurdities which cannot be for a moment entertained. If these "attributes" exist in the manner described by Dr. Burnett, they must include within themselves the nature of self-subsistence; and for that reason must exist abstracted from their own activity (as, without this, the idea of self-subsistence is done away); for it would be absurd to imagine that a self-subsisting principle depended for existence upon its own activity. Dr. Burnett is, therefore, obliged to admit the existence of an *unconscious* consciousness, and a will which has not *volition*, &c.

The same absurdity will follow on a supposition of the mind's materiality; for these attributes cannot include within themselves the nature of self-subsistence: some entity must, therefore, be admitted to exist in which alone they can inhere.

If, then, we have no evidence in support of the existence of mind abstracted from consciousness, perception, &c., it necessarily follows that these attributes must be essential to its existence; and if essential, the mind itself must be the same in nature, consequently immaterial. Indeed, these "attributes" being unable to exist independently of each other, emanate purely from the mind, as the fountain of intellectual life, and thus diverge, as its streams, into these different directions. It is the *mind* which perceives, remembers, combines, compares, and reasons,—which loves, and fears, and hopes; operations which inspire the fullest conviction that the same functions would continue to be exercised in undiminished activity, though all material things were at once annihilated.

That the *mind* is not composed of an "aggregate" "series or collection" of powers, qualities, or faculties, is evident from another view of the absurdity of the supposition. Let us suppose, for instance, the *judgment* to exist alone; it then follows that we must judge without perceiving; and to suppose that the mind can judge without perceiving, is to suppose it to decide upon a subject of which it can have no perception. If, again, we suppose *perception* to exist abstracted from judgment, it will end in the same absurdity; for it supposes the mind to be certain of its own perception, while it is destitute of all judgment, whether it have any perception or not. Or, if we suppose the *will* to stand alone, we must then suppose it to be a will destitute of activity, without a source, and without an object, which is a contradiction.

If, then, the judgment cannot exist abstractedly from the other powers of the mind, and if neither of them can claim any independent state of existence, or be even supposed to stand alone, it follows that all these relative powers must inhere in some common substance, to the nature of which they give a fixed denomination.

As, therefore, this entity must on this account partake of their common nature, and as these attributes cannot be material, nor simply the functions of cerebral structure, it follows, as a necessary consequence, that the human mind must have a positive existence, or, in other words, be an entity. But Dr. Burnett affirms that these attributes "possess no real power over each other," and that is "one reason" why he supposes them "to be separate."

Now, if consciousness and volition, perception and judgment, necessarily imply the existence of an entity, they cannot, by inhering in that entity, either communicate to, or acquire from it, a nature totally opposite to their own, and therefore distinct from it.

Nor can a whole by any means possess a nature of which those powers and properties that are necessary to its existence are entirely destitute. If it can, then consciousness and will, perception and judgment, are not necessary to its existence. If it cannot, then either consciousness and will, perception and judgment, must be materially extended, or the mind must necessarily have a positive existence.

If "*the mind cannot be an entity*," as Dr. Burnett asserts, then perception, judgment, and the other attributes, can only exist on a supposition that they are materially extended, which is impossible; for no two things, which are material, can occupy in one instant the same given portion of space.

Now, if the attributes of the mind exist after the manner described by Dr. Burnett; if, in fact, they "have their root in the motive power of the nervous system," they must be material; and if they are material, they can neither co-exist in the same mental entity, nor operate so diversely and conjointly as we perceive them to do, without occasioning such an interference with one another, as the nature of cerebral matter expressly forbids.

But that these mental powers do and must co-exist, notwithstanding the egregious error of Dr. Burnett in asserting, "*that the individual intellectual faculties may be engaged separately from each other, and from the social faculties*," is evident from the impossibility and absurdity of admitting their separation; and that they cannot be materially extended is the necessary result of their co-existence.

Dr. Burnett is, however, seemingly assured, that his opinions on the philosophy of mind admit of no doubt; and, as if to enhance their importance, he has derogated from the high estimation in which we hold the doctrines of men eminent in this department, by pointing out what he conceives to be the occasion and source of all the discrepancy of opinion which has been expressed relatively to the subject. But in this we charge upon the author either gross ignorance or wilful misrepresentation.

"Mental philosophy," he says, "has had its theory of animal spirits, the doctrine first propounded by Descartes, and subsequently adopted by the school of Locke," (p. 140.) What a mistake! Locke did not adopt—but, on the contrary, rejected the "doctrine propounded by Descartes." Though Descartes exploded the notions of the ancient schools,—that ideas have distinct existences, he nevertheless maintained another doctrine quite unfounded, if not equally erroneous, viz., *that of innate ideas.*

On this point he remarks, in his treatise entitled, "Meditationes Philosophicæ de prima Philosophia," that "the mind, in looking round to extend its knowledge, first finds within itself ideas." "It also finds within itself certain common notions." "Revolving within itself its various ideas, it finds one of a being supremely intelligent;" "attending to this innate idea of Deity," &c.

On the contrary, Locke, in his great and immortal work, the "Essay on the Human Understanding," has discarded all systematic theories; and from actual experience and observation, delineated the features and described the operations of the human mind.

After clearing the way, by *setting aside the whole doctrine of innate notions and principles,* both speculative and practical, the author traces all ideas to two sources, sensation and reflection; thereby overthrowing the doctrine propounded by Descartes, and introducing a new era in mental philosophy.

The unpardonable error committed by Dr. Burnett, with respect to Locke, does not stand alone on the same point. We allude to the reasons which he assigns for difference of opinion on the subject of mind.

His words are:—

"If I mistake not, all the discrepancy of opinion in different writers on the philosophy and physiology of life and mind, and the phenomena peculiar to bodies thus endowed, is to be referred to two great points or errors, viz., 1st, the supposition that all these phenomena result from the action of the same efficient cause in all, subject to degrees of difference in the organization, and 2ndly, that our *perceptions* and *ideas* have *real existences.*"—p. 141.

Taking the two errors which Dr. Burnett here points out, apart from the remarks on Descartes and Locke, we should infer that he actually knew nothing of the works or opinions of these eminent philosophers; and that he had given his attention exclusively to the doctrines of Pythagoras, Plato, and others of the ancient schools; and to Malebranche, Berkley, Hume, Gall, Spurzheim, and others of more modern times.

The doctrine that *our perceptions and ideas have real existences* is not a doctrine advocated by any writer of eminence of very recent date; nor, that we are aware of, in any way acknowledged by the schools of the present day.

It is highly reprehensible to misrepresent, or lapse into an error on a subject of this nature, when, with due consideration, it might have been avoided; but to take up this faulty procedure as a pretext for propounding doctrines the most dangerous, contradictory, and absurd, demands at our hands the severest reprobation.

The doctrines propounded by the ancient schools, that of Descartes, and those of Berkley, Malebranche, Boscovich, and Kant, are far more feasible than a doctrine which, while it denies to mind *a positive existence*, resolves its phenomena *into those of the functions of organic matter*.

This is the position which Dr. Burnett occupies with respect to the subject, as the following quotations will enable us to show:—

"Mind is a mode of action by which the character and qualities of everything around are depicted."—Preface.

"The mind is only, as I shall clearly show, a *mode of action* dependent for its manifestation upon the immaterial spirit of life acting upon a particular organization."—p. 144.

"The mind is no other than a compound mode of action, the effect of the spirit of life upon cerebral matter."—p. 164.

"It has been shown that the manifestation of thought through the brain is strictly dependent on the quantity and quality of the blood supplied to that organ. So that, although the phenomena of the spirit of life which forms the basis of mind, viz., the different modifications of sensation and motion, are inseparable from the brain and the nerves, and the ganglionic system; it does not, therefore, follow, that the immaterial substance causing the phenomena are separate and distinct from that which causes the mental phenomena, or that sensation and voluntary motion, which form the basis of mind, are any other than the spirit of life acting upon the particular combinations of organized matter. Still less does the physiological fact, that sensation and motion, the two fundamental elements of mind, are seated in the cord, and not in the brain, help us to infer that the mind has an independent entity of existence."—p. 151.

Speaking also of the power of sensation, as dependent on the "spirit of life," and the influence which this spirit exerts in contact with matter, Dr. Burnett states that—

"All the higher properties we notice in man, comprehended in the desires, the feelings, the thoughts, and all the high attributes of the mind, are only the more elaborate examples of its modifications."—p. 148.

We have already seen that Dr. Burnett *denies the existence of mind*. We shall now show, that this principle, which raises man in the scale of creation, is to be regarded simply as a mode of action, a quality, effect, or result of organized structure; in short, that the attributes which we regard as an evidence of the existence of an immaterial entity, are the properties or modifications of matter existing under particular conditions.

The phrase "*mode of action*" is one which the author employs without any logical connexion with the notion that *mind* is "*the effect of the*

*spirit of life*" upon "*cerebral matter.*" To speak of the mind being "*a mode of action,*" when it is described as an effect, or result, of the union of a supposed spirit of life with nervous matter, indicates, to say the least, an imperfect and crude mode of thinking, and a desire to mislead and mystify the subject.

If the *mind* be "a *mode of action,*" how is it that the various elements (which in the aggregate constitute our notion of mind, according to Dr. Burnett's views) should be localized to the sentient and motive power of the nervous system? Of the existence of the human mind, we are assured by its operations, viz., in the act of thinking; while its localization to the nervous structure destroys the idea of an independent mode of action, and limits our views of mind to the functions of materiality. If the attributes of mind to which we have previously adverted; if, in fact, the qualities which contradistinguish spirit from matter localize in the nervous structure, it then follows that the nervous structure itself must think. And if mere nervous matter be capable of thinking, thinking must be an essential property of its nature: and, if thinking be an essential property of its nature, no portion of cerebral matter can exist abstracted from it; without admitting this, its essentiality is destroyed.

"It has been shown," says Dr. Burnett, "that the manifestation of thought through the brain is strictly dependent upon the quantity and quality of the blood supplied to that organ." This statement, taken in connexion with others, which limit mind to cerebral or spinal matter, makes thinking essential to its nature, whether in a diseased or healthy state: or whether a portion of the cerebral mass be removed by accident or not. Cases of this kind have occurred in which a portion of the cerebral mass has been separated. Now, if thinking be an essential property of the brain, then either this *thinking* must adhere to some particular part of this divided, or separated portion, or be divided with it. If the former, then that portion of cerebral matter, which is separated from that portion to which this quality adheres, must exist where no thinking can possibly be: and this demonstrates, that thought is not essential to its nature; that its "*mode of action*" cannot be that of thinking, nor its phenomena that of thought. But if we suppose thinking to be divided with the portion which was separated from the brain, it can then exist in no part of this divided portion. For to suppose *a divided quality* or "*mode of action,*" to exist by inherence in two portions of divided nervous matter, is to suppose it to be dividable, or *to exist* and *not to exist* at the same time. As, therefore, the idea of a divided thinking includes a contradiction, it necessarily follows, that, in either case, thinking cannot be essential to nervous matter.

Moreover, the brain is an extended substance; and if it be capable of thinking, thinking must be either as *extensive* as its dimensions, or be confined to some particular part. In the first case, if the *actions* of this diffused principle were to be directed to the central point of this extended substance, thinking must operate in opposite directions, which opposition in its directions will at once prove the diversity of its nature, and, consequently, destroy the unity and simplicity of its existence. For if a *simple* action of the mind can arise from a principle which is necessarily extended and diffused, this action must derive its being from an energy which cannot equally contribute to its existence. But if this thinking be confined to some particular part of the brain, then it follows that cerebral matter is different from itself, because one part only is supposed to be capable of thinking, and the other part not; which ends in this contradiction, that the brain thinks and does not think at the same time.

To suppose, then, that thinking is as extensive as the dimensions of the brain, is to suppose that an action of the mind, or, according to Dr. Burnett's theory, a "mode of action," can arise from a cause which can give it no existence; while, on the supposition that the property of thinking, or its "mode of action," is confined to some particular part of the brain, the brain is made capable of thinking, and incapable at the same time; the rational conclusion therefore is, that the brain cannot think, nor thinking be its "mode of action."

If "the mind is no other than a mode of action," to what does this "mode of action" apply? It cannot be said to apply to mind, because the distinguishing principle of that to which the "action" refers, is wanting. A "mode of action" may appertain to anything in the physical world; but here it stands disconnected, and is, consequently, without rational interpretation.

If the "mind" be a "mode of action," then, what is the mind? Is it the action, or is it the mode of its performance? If it be the action, then action is mind; if the manner by which it characterizes itself, then the manner in which it does so characterize itself must be the mind also. But *where* is the mind? Dr. Burnett has not included it in his definition. The phrase "mode of action," does not embody the terms of a definition of mind. The *predicate* is imperfect—*desunt cætera.* This imperfect attempt proves that the author intended to give a definition that should rightly convey a correct notion of mind; but in this he has signally and lamentably failed. Let him not attach blame to us: for we have acted friendly in pointing out to him the monuments of present, that he may avoid the sources of future failure.

Had Dr. Burnett included the *act of thinking* in his definition, the same objection would not hold; for, after all, thinking is but an action, while the phenomena of thought at once directs us to the immaterial agency on which it depends.

If thinking be an essential property of the human brain (which the views of Dr. Burnett unquestionably implies), then thinking and cerebral matter must be co-existent; for the latter could never exist without the former, seeing it is presumed to be an essential property of it. But since thinking is only an action, it is absurd to imagine that a mere action can co-exist with, or be an essential property of that which can perfectly exist without it.

The same absurdity follows, on the supposition that *mind* is "*the effect of the spirit of life upon cerebral matter.*" It has been already demonstrated, that a "spirit of life" does not exist, or, in other words, that "life" has not an abstract *positive existence.* Now, that which has no *positive existence* can produce *no effects.* How, then, can mind be the "effect" of a "spirit of life" upon cerebral matter? Every effect must have a cause, and a cause adequate to its production. To suppose, then, *mind* to be an *effect* of a "spirit of life" operating upon cerebral matter, is to give *being* to an *effect* abstracted from the existence of a cause, necessary to the existence of such effect. But *life* is not *mind,* nor *vice versa*—how, then, can such a spirit as life is supposed to be, produce *mind*—an entity, whose existence is known only by its operations—viz., in the act of thinking? If it can, then this thinking is essential to life and nervous matter; and, if essential, life and nervous matter must not only think always, but think always in the same direction. To suppose otherwise, is to suppose that life and nervous matter are capable of producing *effects,* which are contrary to their own *effects,* or that the necessary effects of life and nervous matter are contrary to the necessary effects of life and nervous matter, which is a contradiction.

If mind thus be dependent on life and cerebral matter, thinking cannot exist, or extend beyond the compass of this cerebral matter. That the mind can extend its operations not only beyond the dimensions of the brain, but even beyond the limits of the globe, is a point which claims to be self-evident, and therefore requires no proof. We have then, in this case, a clear idea of the mind acting, where neither a "spirit of life" nor cerebral matter is supposed to exist; and if the mind can extend its operations beyond the limits of inanimate matter, it undeniably follows that thinking cannot be the "effect" of a "spirit of life" upon cerebral matter, nor be dependent upon their union for its existence. Again, if mind be the "effect" of a "spirit of life" upon "cerebral matter;" or, if it result from their union, then a *potential or virtual energy* must exist in the cause as it does *formally* in the effect:

for if this be not conceded, we must suppose life and cerebral matter to be *capable* of producing an effect which they have no power to originate, or that an effect is in being, independent of a cause to produce it, which is a contradiction.

As, then, this potential or virtual energy must necessarily inhere in cerebral matter, in order to the production of mental action as its effect or result; and, as cerebral matter, whether acted upon by a "spirit of life" or not, is an extended and dividable substance, it necessarily follows that no such energy can reside within it; and, consequently, that no such action can result from it; and, therefore, "cerebral matter," as acted on by a "spirit of life," can have no such energy resident within it to produce, and can have no such action as its effect or result.

In the quotation previously given, Dr. Burnett speaks of "sensation and voluntary motion, which form the basis of mind," in a way that betrays great lack of knowledge on the important relation which sensation bears to the mind. It is no doubt owing to the neglect of more closely investigating this point—viz., the relation existing between thought and sensation since Locke first claimed for it the attention which his predecessors had failed to do, that mental philosophy has been altogether excluded from the rank of an inductive science.

Our analysis of Dr. Burnett's work having already exceeded the usual limits, we cannot do more on the present occasion than direct attention to this important point.

We are thus precluded from adverting to other matter contained in the work; but we have the satisfaction of knowing that we have fully exposed the greater part of the fallacious hypotheses which the author endeavours to establish.

---

Since the publication of our last journal, Dr. Burnett has written to us in rather angry terms, complaining that his views have not been fairly represented. He says, "my theory has been most unjustly stated in your review; and I am justified in demanding that it shall be put right." "The most essential part of the title-page of my work—viz., this—showing the real existence of two very distinct kinds of entity, which unite to form the different bodies that compose the universe, organic and inorganic, by which (union) the phenomena of light, heat, electricity, motion, life, mind, &c., are reconciled and explained, is entirely omitted."

"It is not my object to prove that heat and electricity are distinct entities co-ordinate in rank, and of an immaterial or spiritual nature,"

as we stated, "but to prove that light, heat, &c., *are not entities, but modes of action resulting from the union of spirit with matter.*"

What an apology for a theory so unfounded! The language now employed is but an exponent of the same doctrine, under a different phraseology! while the *title-page* alone more than confirms the accuracy of our statements.

The author informs us that it is his object to prove that heat, electricity, &c., "*are not entities,*" "*but modes of action,*"—then what becomes of the meaning of the "*title-page?*" Are contradictions to pass current in a work professedly designed to revolutionize science and philosophy? We never met with a collocation of words—"*the real existence of two very distinct kinds of entity*"—so glaringly solecistical, but which the author evidently employed to prevent the possibility of his meaning being mistaken. "*Real existence,*" is opposed to *fictitious,* not imaginary, true, genuine. "*Very distinct kinds,*" means, different the one from the other, in an eminent degree—the particular nature of each not similar. "*Entity*" signifies something which really is—a real being. Now, the two entities here referred to, are heat and electricity; and yet Dr. Burnett gravely tells us that *his object is to prove that they are not entities.*

The author speaks of these entities at page 21, as "having been brought into a real and independent existence,"—"these distinct kinds of entity," &c. At page 36, he says: "From the argument I have already adduced, and the proofs I am about to give, I do think that *such substances have an independent immaterial existence which may be logically proved.*" At page 10, he remarks: "*that there are two very distinct and characteristic kinds of substances, both alike as entities, but totally different and opposite in their nature*"—(the author's own italics.)

It is evident from these quotations (and many others might be given) that Dr. Burnett does endeavour to establish the *positive existence* of heat and electricity, as well as the hypothesis, that they are "*modes of action, resulting from the union of spirit with matter.*"

That mere assertions and opinions are not logical proofs, we shall proceed to show. If a demonstration of the truth of the theory which Dr. Burnett here propounds could be given, wherefore use such a term as "modes of action?" What reader can divine its import in the sense in which it is employed? We condemn it as a faulty, vicious, and misleading form of expression. In a note at page 11, the author has certainly defined his meaning, but on no subsequent occasion is it referred to. It is this:—"*By phenomena, or modes of action, I mean such effects as are produced by the application of one or both of the uncombined entities to created matter.*" If "modes of action" are "effects,"

why has this definition not been adhered to? Why has the author, in the use of that term, in the case now under our consideration, made heat, electricity, &c., to have an independent existence? By the supposition that heat and electricity are "modes of action," a being is given to an effect, abstracted from the cause which produces it. No "action," in the sense of being an "effect," can apply to a "mode;" for this mode is itself dependent, and precludes, therefore, the idea of any further dependency. Thus, if heat and electricity are "modes of action," then these actions are the effects or results of the modes, which is absurd, and directly contrary to the definition, that a mode of action is an effect of the union of spirit with matter.

The very form of expression, then, in which Dr. Burnett denies the accuracy of our statements, will enable us to expose, in no slight degree, the futility of the charge which he has made against us.

The language, "*modes of action resulting from the union of spirit with matter*," necessarily presupposes the abstract positive existence, or, in other words, the *previous* existence of spirit and matter.

If heat and electricity *result* from the "union of spirit with matter," it is evident that they could not have existed previously to the time when this supposed union took place, from which they are said to result. Here is a dilemma: we have the previous existence of heat and electricity necessarily supposed; at the same time it is asserted that they did not exist, and that they are the result of their union with matter, as necessary to their own existence afterwards.

If heat and electricity result from the *union* of spirit (and heat and electricity are the spirit here intended) with matter, then their existence is thus ascertained distinct from matter. And if heat and electricity result from the union of spirit with matter, matter and spirit must have existed antecedently to their union with each other. And if both matter and spirit did exist prior to their union with each other, it then follows that heat and electricity do not depend for their existence upon *their union* with matter. And if this dependence be taken away, it must also follow that heat and electricity (or "spirit," as Dr. Burnett terms it) may as well exist after their separation from matter, as they did before their union with it. Either this "spirit" must have existed prior to its union with matter, or it must not. If it did, heat and electricity cannot be "modes of action," or results of the union of spirit with matter; if not, they cannot be the result of that union.

The only possible ways in which heat and electricity can be supposed to result from the "union of spirit with matter," must be from matter as a *substance*, or from *some peculiar arrangement* or *alteration* of its chemical or molecular constitution.

But in this case, the idea of "spirit" entering into an alliance with matter, before heat and electricity can become their offspring, is not included. Dr. Burnett has therefore no advantage to gain by the supposition. We have excluded the term "modes of action" from the previous proposition, for three reasons. 1. Because the "action" of a "mode" is physically impossible, and logically absurd. 2. Because we agree with Locke, when he says, "modes contain not within themselves the supposition of subsisting by themselves, but are considered as dependencies on, or affections of substances." 3. Because Dr. Burnett himself states that "by modes of action," he means "such effects" as those which take place in the case now under consideration.

Bearing in mind that Dr. Burnett now avows heat, electricity, &c., *not to be entities,* but modes of action, altogether dependent upon the "*union of spirit with matter,*" we subjoin the following, to disprove the hypothesis contained in the former quotation :—

"It is," states Dr. Burnett, "from the great difference in the visible appearance of the heavenly bodies that I am led to suppose the immaterial substances have not only different qualities, and also relative degrees of power, *but that they possess also a power of occupying all space.*"—p. 89.

Here, then, we have an hypothesis which regards heat, electricity, &c., as possessing a positive existence; or, in the words of the title-page, "showing the real existence of two very distinct kinds of entity"—"*where no matter is supposed to be*"—an hypothesis which makes those supposed "spirits," "modes of action," or "results," to extend themselves *beyond their own existence*—to act, where from their absence they can have no power of acting—and that they are *present,* as "modes of action," or results of the union of spirit with matter, and yet *absent* in space where no matter is, at the same time. It therefore follows, either that they do occupy all space, or that they do not result from their union with matter. That they cannot occupy all space, nor result from their union with matter, we have before disproved; and as a contradiction is ever inadmissible, we conclude, that Dr. Burnett has completely failed either in affording the slightest pretext in support of his own hypotheses, or in support of the charge of partiality, which he has attempted to allege against us.

In taking our leave of Dr. Burnett, we would observe that there are certain points, ever necessary to be borne in mind by all aspiring to become authors, viz. :—

1. That the doctrine sought to be established in philosophy, be supported by such evidence—that is, by arguments, facts, and principles—which will admit no doubt of its truth.

2. That no other than the common grounds of reasoning be entered upon.

3. That a proposition once demonstrated to be true, no contrary reasoning can demonstrate it to be false, *i. e.*, logically fictitious.

Attention to these simple rules, would prevent much unnecessary trouble and annoyance both to authors and reviewers.

